# Renal Infarction as the First Manifestation of Undiagnosed Atrial Fibrillation With Coexisting Left Atrial Thrombus: A Case Report and Review of the Literature

**DOI:** 10.7759/cureus.53772

**Published:** 2024-02-07

**Authors:** Ahmad Damlakhy, Khaled M Harmouch, Zohaib A Khan, Nomesh Kumar, Anas Abdel-qader

**Affiliations:** 1 Internal Medicine, Detroit Medical Center/Sinai-Grace Hospital/Wayne State University, Detroit, USA

**Keywords:** abdominal pain, thromboembolism, intracardiac thrombus, paroxysmal atrial fibrillation, renal infarction

## Abstract

Acute renal infarction, presenting with nonspecific symptoms, such as abdominal pain, nausea, vomiting, and hematuria, can lead to delayed diagnosis due to similarities with other medical conditions. Computed tomography with IV contrast is used to diagnose renal parenchymal infarction, treated through surgical, percutaneous interventions, and anticoagulation therapy. Investigation for the infarction source is crucial, particularly in the absence of prior cardiac issues, necessitating heart rhythm monitoring and an echocardiogram to evaluate paroxysmal atrial fibrillation (PAF) and intracardiac thrombus, respectively. Renal infarction may elevate blood pressure due to renin release, recommending medications like angiotensin-converting enzyme inhibitors/angiotensin receptor blockers. We present a case of renal infarction due to PAF with a concomitant intracardiac thrombus.

## Introduction

Renal infarction is a result of an acute disruption of blood flow in the renal artery and its distributive branches. The disruption can occur secondary to either an embolic source from the heart, a hypercoagulable state, or intrinsic or extrinsic damage to the kidney and its vasculature [1]. In regards to embolic sources, a common cause is atrial fibrillation (AF), with multiple studies showing that AF has accounted for as high as 48% and 61% of cases of renal infarctions [2,3].

Cases of renal infarction are important to highlight as there is a need to include renal infarction in our differentials while dealing with patients presenting with the right symptoms. Various studies have shown that the majority of cases present with abdominal and flank pain as well as with nausea and vomiting [2-4]. In the constellation of symptoms, most patients are initially diagnosed with either urethral stones or gastrointestinal issues. Renal infarctions were not among the initial differential diagnoses in the 42 cases, despite them actually being renal infarcts [4]. This often leads to delayed diagnosis with resultant complications.

Early diagnosis and treatment are needed as the majority of patients presented with acute kidney injuries, with some cases requiring dialysis [1,5]. As noted, cases of renal infarctions can additionally present with an uncontrolled hypertensive crisis requiring a multifaceted approach to treatment [6].

Despite the severity of the disease and the consequences of a missed diagnosis, renal infarcts remain highly treatable conditions. The successful treatment and resolution of resultant acute kidney injury with both conservative and thrombolytic therapy have been demonstrated, emphasizing the importance of timely diagnosis and treatment [7].

## Case presentation

A 53-year-old female with a significant past medical history of hypertension presented to the emergency department with the chief complaint of left lower quadrant abdominal pain one day prior to presentation. The pain had a sudden onset, was stabbing in nature, rated 8 out of 10 in severity, and was accompanied by nausea and vomiting of undigested, yellowish food. She denied hematemesis, constipation, diarrhea, melena, or hematochezia. The review of systems was unremarkable, and she denied chest pain, shortness of breath, palpitation, cough, sputum, dysuria, hematuria, fever, or chills.

**Figure 1:**
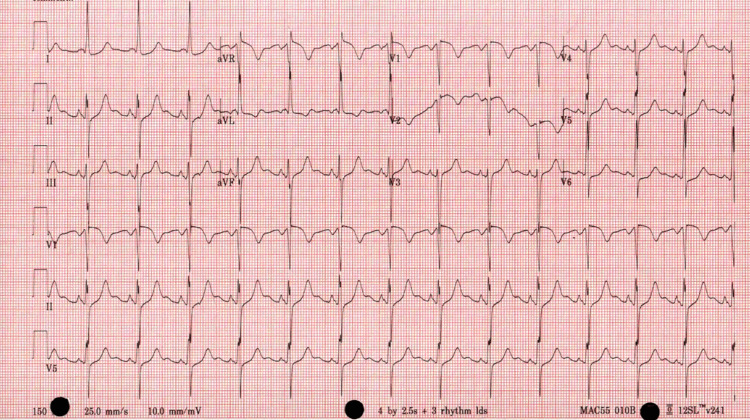
Electrocardiogram showed normal sinus rhythm

On presentation, vital signs revealed elevated blood pressure at 191/119, heart rate of 85, temperature of 36.8°C, respiratory rate of 18, and O2 saturation of 98% on room air. Physical examination showed tenderness over the left lower quadrant abdomen and left costovertebral angle tenderness, while the cardiovascular exam was unremarkable. Blood workup revealed hypokalemia with potassium at 3.1 mmol/L (normal range: 3.5-5.1 mmol/L), chloride at 91 mmol/L (normal range: 98-107 mmol/L), sodium at 133 mmol/L (normal range: 136-145 mmol/L), creatinine at 1.07 mg/dl (normal range: 0.6-1.2 mg/dl), lactate dehydrogenase (LDH) at 632 units/liter (normal range: 140-270 units/liter), and mildly elevated liver function tests (alanine aminotransferase = 71; aspartate aminotransferase = 86). Total bilirubin and direct bilirubin were within normal limits, and urinalysis showed no signs of infection or RBCs in urine. The electrocardiogram (EKG) on presentation indicated normal sinus rhythm with no ischemic changes, as shown in Figure 1, and chest and abdominal X-rays were unremarkable.

**Figure 2:**
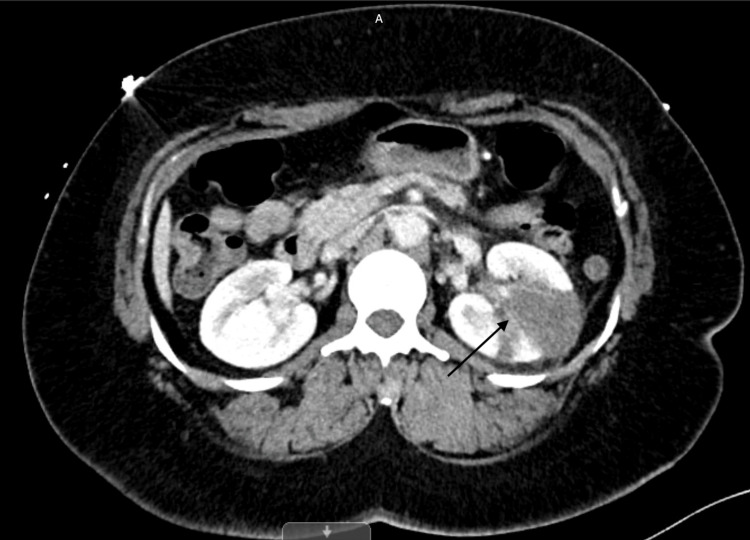
CT of the abdomen and pelvis with IV contrast Black arrow showing wedge-shaped infarction in left kidney.

During hospitalization, despite administering antiemetics and antihypertensive medications (hydrochlorothiazide 25 mg daily and nifedipine 60 mg daily), the patient's blood pressure remained uncontrolled. A CT of the abdomen and pelvis with IV contrast revealed a wedge-shaped hypodensity of the left kidney, suggesting focal acute pyelonephritis versus renal infarction, as depicted in Figure 2. Subsequent CT angiography of the thorax, abdomen, and pelvis showed left renal infarction with intact left renal artery. Considering the lack of infection symptoms and normal urine analysis, pyelonephritis was not at the forefront of our differential diagnosis.

**Figure 3:**
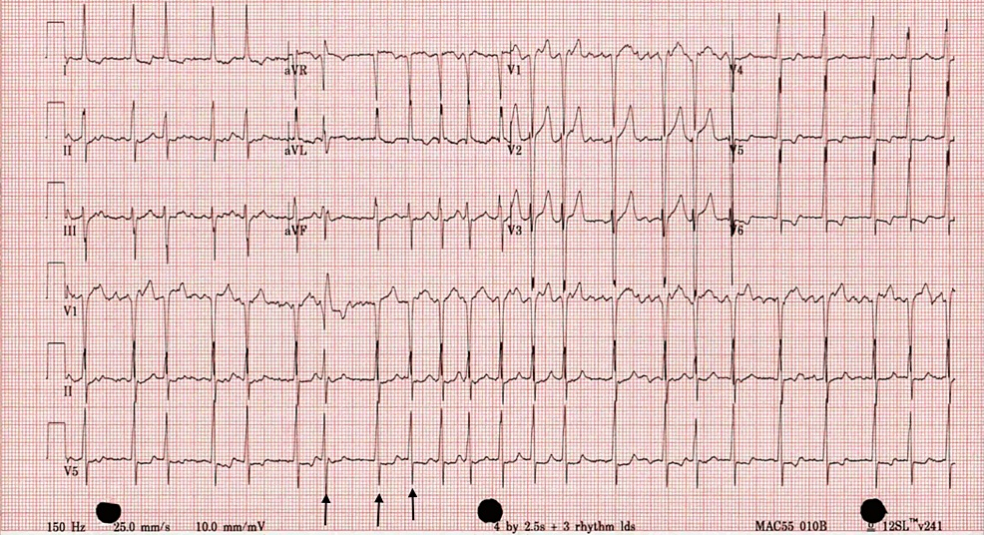
Electrocardiogram Black arrows in lead V5 show irregular QRS complexes in the absence of P waves.

**Figure 4:**
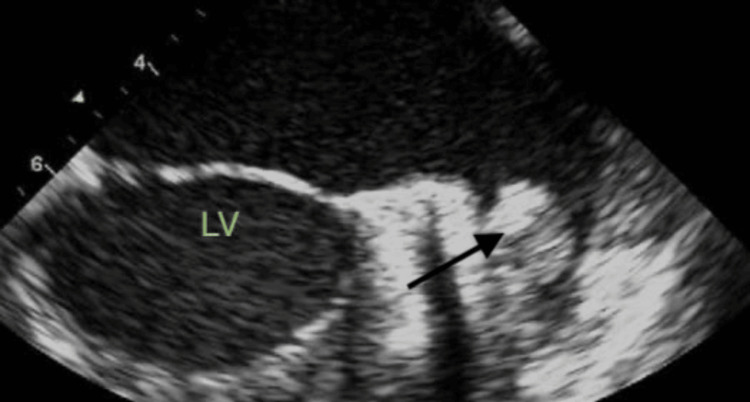
Transesophageal echocardiogram Black arrow showing thrombus in the left atrial appendage. LV: left ventricle.

On the second day of hospitalization, the patient's symptoms resolved and blood pressure normalized after starting lisinopril. However, on telemetry, the patient exhibited a heart rate of 136, and a 12-lead EKG revealed atrial fibrillation with rapid ventricular response (RVR), as shown in Figure 3. IV metoprolol 5 mg was administered, reducing the heart rate to 100 beats/minute, but the rhythm remained irregular. A transthoracic echocardiogram (TTE) showed a normal ejection fraction with no evidence of intracardiac thrombus. The patient was started on a heparin drip, cardiology was consulted, and transesophageal echocardiography (TEE)-guided cardioversion was planned. TEE revealed a left atrial appendage thrombus, as demonstrated in Figure 4, leading to a decision to defer cardioversion at that time. The patient was switched to oral anticoagulation with Eliquis 5 mg twice daily, in addition to rate control with metoprolol 50 mg twice daily.

Notably, vascular surgery and interventional radiology were consulted for renal infarction, and anticoagulation was recommended without surgical intervention. The patient was discharged on oral anticoagulation and rate control medication, with a plan to follow up with cardiology.

## Discussion

Acute renal artery thromboembolism is a rare but significant cause of severe renal damage. Due to its infrequency and non-specific symptoms, diagnoses are often delayed. The primary association is with cardiac issues, especially atrial fibrillation. Clinical presentations include varied symptoms like abdominal pain, nausea, vomiting, hematuria, proteinuria, and fever [5]. For partial renal infarction, a high clinical suspicion is crucial. Key indicators involve a history of cardiac arrhythmia, abdominal/flank pain, fever with leukocytosis, and elevated LDH levels [8]. In our patient who experienced renal infarction without any prior cardiac arrhythmia history and presented with a normal sinus rhythm, it is essential to utilize intensive EKG monitoring to pinpoint the source of the infarction.

Precise diagnosis is vital to protect renal function. In cases of severe and unexplained abdominal or flank pain, along with microscopic hematuria, increased LDH, and leucocytosis, consider renal artery infarction. LDH, a highly sensitive indicator, is typically five times higher than normal in renal infarction. While elevated LDH can occur in conditions like hemolysis, myocardial infarction, and mesenteric ischemia, physicians should explore common risk factors such as previous thromboembolic events, aorta interventions, cardiac disease, and atrial fibrillation [9].

CT scan plays a crucial role in the assessment and treatment of primary renovascular disease. Non-enhanced CT proves valuable in illustrating renal hemorrhage, renal parenchymal or vascular calcifications, and masses. Contrast-enhanced CT is indispensable for recognizing renal abnormalities resulting from the vascular process, such as renal infarcts. If a non-enhanced CT reveals no urinary calculi, contrast-enhanced CT of the abdomen may be necessary for early detection of renal infarction. CT, being noninvasive, sensitive, and capable of identifying segmental lesions, emerges as the optimal method for diagnosing renal infarction [10].

In cases where renal infarction is suspected, it is recommended to undergo both TTE and TEE for a comprehensive assessment of potential cardiac sources of emboli. This evaluation encompasses various aspects, including the presence of mural thrombus, assessment for valvular pathology, and consideration of congenital heart conditions like atrial septal defect or patent foramen ovale, which might contribute to paradoxical embolism. In our case, TTE showed no issues, but TEE revealed a thrombus in the left atrial appendages. In instances of cryptogenic embolism in a patient exhibiting sinus rhythm and lacking cardiac abnormalities, it is crucial to consider the potential coexistence of paroxysmal atrial fibrillation (PAF). The utilization of Holter monitoring may facilitate the detection of concealed PAF in individuals experiencing cryptogenic embolism [11]. Additionally, a thorough investigation is warranted for any infarction occurring in other organs like the spleen, bowel, and liver.

In the context of renal infarction, it is crucial to reinstate renal blood flow to prevent potential renal failure. Different approaches such as surgical intervention, percutaneous intervention, thrombolytic therapy, and anticoagulant therapy are available for managing renal arterial embolism [8]. Usually, revascularization is most beneficial for patients under certain conditions: for complete arterial occlusion lasting less than six hours, a solitary kidney, or a significant decline in kidney function. In our case, given that the laboratory results indicated normal kidney functions, symptoms manifested only a day ago, the presence of PAF that requires anticoagulation, and the CT scan unveiled a confined infarct area, we opted for conservative therapy with anticoagulation instead of percutaneous intervention or thrombolytic therapy in this particular case.

It is important to note that many patients develop high blood pressure readings after renal infarction, even in the absence of a history of hypertension. This phenomenon can be explained by the release of renin. It is preferable to manage blood pressure with angiotensin-converting enzyme inhibitors (ACEIs)/angiotensin receptor blockers (ARBs) in the absence of contraindications, such as acute kidney injury or hyperkalemia, as these medications work on the renin-angiotensin-aldosterone system (RAAS). In our case, the patient initially had high blood pressure readings that were uncontrolled after initiating hydrochlorothiazide and nifedipine. However, the introduction of lisinopril significantly improved blood pressure [12].

## Conclusions

Acute renal infarction is an uncommon condition. In the absence of previous cardiac issues, an investigation for PAF with heart rhythm monitoring and an echocardiogram to look for a potential cardiac source of embolus should be done. Those patients might develop high blood pressure readings, either in the presence or absence of a history of hypertension, which can be attributed to the release of renin. Managing blood pressure with medications that work on the RAAS, like ACEIs/ARBs, helps control it.
